# Some Cases of Osteomyelitis

**Published:** 1896-06

**Authors:** W. R. Blailock

**Affiliations:** McGregor, Texas


For the Texas Medical Journal.

### SOME CASES OF OSTEOMYELITIS.

BY W. R. BLAILOCK, M. D., M'GREGOR, TEXAS.

Read before the Section on Surgery, Texas State Medical Association, at Fort Worth, April 28th, 1896.

OSTEOMYELITIS is of microbic origin, and the so-called spontaneous variety occurs almost exclusively in young persons, whose osseous development is not completed. Traumatic and tubercular osteomyelitis may occur while the bones are developing, or after their full development.

Two of the cases that I shall report may be classed with the so-called spontaneous variety, while the other may be considered of tubercular origin.

Case 1.—Male, aged 5 years; previous health good; family history gave no evidence of syphilis nor tuberculosis.

July 1.—Jumped from a wagon; stuck a thorn in left heel, which was pulled out. Some inflammation ensued, and a few drops of pus formed, which escaped through a small opening in track of thorn.

July 12.—Suffering from intense pain in left leg, any movement of which caused him to cry as though a knife were being thrust into the leg.

The heel was apparently well, and a small incision did not reveal the presence of pus. Temperature 103° F.; tongue coated and parched; bowels constipated.

July 13 and 14.—Pain and elevation of temperature continued.

July 15.—Right tibia painful; worse at night.

July 20.—Both legs intensely painful and swollen.

July 27.—A large fluctuating abscess on anterior middle part of left leg, and opposite line of lower third with middle of right leg. Pain had greatly abated. The pus was sanious and considerable in quantity; all pain subsided after its evacuation, but it continued to escape in small quantity. In the course of three months a small piece of necrosed bone passed from right leg, after which all discharge ceased in that leg.

December, 1889.—A sequestrum three inches long, occupying the anterior portion of left tibia was removed. A fairly strong involucrum had formed, but there was some arching forward of the tibia, owing to neglecting to support the leg by a suitable splint while the involucrum was forming.

All discharge of pus ceased in the course of a month, and that leg has given no further trouble.

July, 1895.—The right tibia became painful in its upper part with a very tender area near the junction of the epiphysis with the diaphysis, but no suppuration followed. The boy is now in good health.

Case 2.—Male, aged 18; previous health good; weight 180 pounds. Tuberculosis had caused the death of an uncle and two aunts on father's side.

July, 1893.—Was working with a threshing machine; sleeping on the ground at night. Had a chill, followed by fever, and a pain in right lung, which the attending physician pronounced pneumonia.

Very soon pain in or near the left knee set up, severe at first, but soon subsided to a great extent. Was treated for rheumatism.

I saw him two months after commencement of illness. Bowels had not moved for three or four days. Complained of pain in the back, which was greatly intensified by moving the body. Pressure over fifth lumbar vertebra showed marked tenderness. Left knee and thigh swollen and somewhat tender. Knee joint could not be straightened nearer than to about 45°. A straw-colored fluid was aspirated from the joint, but no pus found. On introducing the needle above the joint the bone was found uncovered and rough for several inches, especially on the inner side of the lower end of femur.

September 3.—Made an incision about four inches in length on inner side of thigh. Found a quantity of thick, yellow fluid, which had burrowed beneath the periosteum, separating it from she bone on the inner and lower side for a distance of five or six inches. On the under side of the bone, near the lower epiphiseal line was an opening into the bone, near which, lying loose in the fluid, was a lump of caseous material as large as the end of the thumb. An opening was made on the inner side of the bone, and the entire medullary portion found diseased and broken down for a distance of nearly five inches. The diseased bone was scraped and chiseled away, the cavity flushed with bichloride solution 1 to 1000, dried or wiped out with gauze, dusted with iodoform and packed with decalcified bone chips. The bone chips were taken from an alcoholic solution of bichloride of mercury, 1 to 500, carefully dried between strips of gauze; washed in alcohol, and again dried. An opening had been previously made on the outer part of the thigh, connecting with the bone on the under side, through which a drain tube was passed. The incision was carefully sutured with cat gut, approximating the bone and surrounding soft parts as nearly as possible. An anterior splint was placed over the femur, an antiseptic dressing applied, and the leg slightly elevated. Pain and fever subsided, appetite improved, bowels acting well.

Four days after the operation, that part of the dressing into which drainage tube discharged was removed, and found saturated with a yellowish fluid, but no pus. Twelfth day, entire dressing was removed, and incision found healed throughout. The drain tube continued to discharge a small amount of the same yellow fluid, but it was not the least bit offensive. Peroxide of hydrogen was thrown in through the drainage tube, only one drachm of which could be put in by ordinary force at a time, after which iodoform emulsion was thrown in, and the leg redressed as before. After a few treatments of this kind the tube was removed, but a slight discharge continued.

The knee joint remained swollen, flexed and tender; it was again aspirated but only a straw-colored fluid was found. Galvanism was tried, a copper electrode being placed to the sole of the foot, to which the negative pole was connected, the knee completely surrounded by copper gauze, covered with cotton cloth, to which the positive pole was connected. The current was started with eight cells, and gradually increased to eighteen, where it was maintained for fifteen minutes. This treatment was continued three times a week for four or five weeks, when it was found that the swelling was greatly reduced and the leg could be nearly straightened without causing pain.

October 6, 1894, a small sequestrum, about two inches long, occupying the under outer part of the femur, opposite where the bone chips were put in but not connecting with that part of the bone, was removed. After this all discharge ceased. The young man is now, April, 1896, in good general health and has perfect use of the limb.

Case 3.—Male, aged 36; could elicit no history of syphilis, tuberculosis, nor cancer. When eleven years old, while binding wheat, was seized with a violent pain in the left hip, took a bath by jumping into a creek, felt better. Pain returned at night, continued several days, accompanied by high fever. Some time afterward an abscess, formed on outer aspect of the thigh, bursted spontaneously, since which time has had a constant discharge of pus.

March 9, 1896, found him sallow and emaciated, knee anchylosed. A line of cicatrices extended along the outer side of the thigh, from about three inches above the knee to a few inches below the level of the smaller trochanter. Near the middle of this line of cicatrical tissues was a fistular opening, discharging a most offensive sanious pus. When he lay on his back the pus flowed more freely than while standing. For the last few months much blood had been lost through this opening.

An effort was made to cleanse the leg; a constrictor placed high up around the thigh and an incision carried down to the bone through the cicatricial tissue. On a line two inches or more above the fistulous opening a cloaca was found. Below this, a distance of about one and one-half inches, the bone was covered with periosteum; then for a distance of three or four inches the entire shaft of the bone was bare and of a dark color. An involucrum had formed above and the dead bone was firmly wedged above and below, the only escape being the cloaca previously described situated at the summit of the dead bone. When an opening was made into the cavity of the dead bone, a most horrible odor was emitted.

The sequestrum was removed by breaking down with a chisel and mallet, the cavity scraped and chiseled out as thoroughly as practicable, which was none too good, as osteosclerosis had taken place all around; the upper part cauterized with a Paquelin cautery point, to stop bleeding, which was disposed to be troublesome, the cavity washed out with bichloride solution, 1 to 1000, and packed with iodoform gauze.

March 13.—Bad odor emitted from the dressing, which was saturated with pus. Had been eating and sleeping well since operation. When the gauze was removed from the cavity in the bone a most alarming hemorrhage ensued. Placed a constrictor around the thigh and compressed the femoral artery, but the bleeding continued. A strip of gauze was now packed into the upper part of the bone cavity and bleeding ceased. I realized at this juncture that an amputation in the first instance would have given less trouble, as well as the patient a better chance for his life.

March 15.—Pulse, 90; temperature normal; had taken concentrated food and plenty of whisky for past 40 hours; wound very offensive. Did not attempt to remove dressing, which had been kept saturated with corrosive sublimate solution since the alarming hemorrhage two days before, but saturated it with Platt's chlorides and covered the whole with two thicknesses of cloth, also saturated with chlorides.

The thigh was now amputated as high up as possible without making a hip joint amputation. Perhaps not more than two ounces of blood was lost from the proximal end, which was fortunate, as he had no more to spare.

April 5.—Flaps nearly entirely healed, discharging about 2 or 3 drachms of pus in 24 hours; appetite good; is sitting up part of the day with every prospect of recovery.

April 16.—Is walking on crutches and improving rapidly.

Case 1 is interesting as an instance of multiple osteomyelitis, in which the two tibia alone were involved. The pus that formed around the track of the thorn in the heel was doubtless the origin of the bone leosions. The case has other features of interest. It shows what may be looked for if the expectant plan of treatment is followed. Purgatives, opiates and tonics were given, but if the bone had been opened early and the diseased area scraped away and properly disinfected, the result might have been quite different. Eight or ten years ago, however, surgery of the bones was not so well nor so generally understood as it is now. The slight deformity of the leg due to arching forward of the tibia might have been prevented had a suitable splint been worn during the period when the involucrum was forming, but as I did not see him during this time, can not censure myself for it.

Case 2 is of especial interest. It resembled rheumatism so closely that it was treated for a period of two months for that disease. The family history, short duration of severe pain, absence of pus and presence of evidences of *caseation* point to tubercular osteomyelitis.

Other points of interest are (1) the success attending the use of decalcified bone chips. (2.) The adhesion of *paraperiosteal* tissue over such an extensive area of bone that had been separated from the periosteum. (3.) The removal of a small sequestrum from the opposite side from which the bone chips were introduced more than a year after the operation, and finally (4) the prompt benefit arising from galvanism in the manner described, the removal of effusion from the joint and complete restoration of the limb.

Case 3 shows what unaided nature will do. For twenty-five years this man had carried a sequestrum involving the entire shaft of the femur, and had been able to labor on a farm up to a few months ago. Osteosclerosis had prevented the absorption for a number of years of a most intensely stinking pus. This same hardened bone prevented the first operation from being as thorough as it should have been. Considerable time was consumed in removing the sequestrum, and in endeavors to chisel and scrape the hard bone down to an even surface, so much, indeed, that it was unsafe to prolong the operation at that time.

